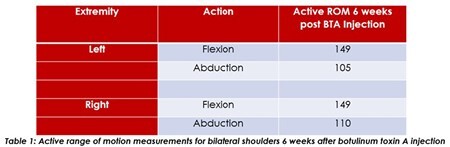# 780 Usage of Botulinum Toxin a in Acute Burn Care: A Case Study

**DOI:** 10.1093/jbcr/irae036.321

**Published:** 2024-04-17

**Authors:** Ariel J Rodgers, Sarah Barth, Lily DuRose, Irma D Fleming, Giavonni M Lewis, Callie M Thompson, Christopher R LaChapelle

**Affiliations:** University of Utah, Salt Lake City, UT; University Of Utah Burn Critical Care, Salt Lake City, UT; University of Utah Health, salt lake city, UT; University of Utah, Salt Lake City, UT; University Of Utah Burn Critical Care, Salt Lake City, UT; University of Utah Health, salt lake city, UT; University of Utah, Salt Lake City, UT; University Of Utah Burn Critical Care, Salt Lake City, UT; University of Utah Health, salt lake city, UT; University of Utah, Salt Lake City, UT; University Of Utah Burn Critical Care, Salt Lake City, UT; University of Utah Health, salt lake city, UT; University of Utah, Salt Lake City, UT; University Of Utah Burn Critical Care, Salt Lake City, UT; University of Utah Health, salt lake city, UT; University of Utah, Salt Lake City, UT; University Of Utah Burn Critical Care, Salt Lake City, UT; University of Utah Health, salt lake city, UT; University of Utah, Salt Lake City, UT; University Of Utah Burn Critical Care, Salt Lake City, UT; University of Utah Health, salt lake city, UT

## Abstract

**Introduction:**

Burn scar contractures over joints can be challenging for everyone, from patient to therapist to burn surgeon. They can be managed in a multitude of ways; however, the use of botulinum toxin A (BTA) in acute burns has not been described. We describe a case report introducing the possibility of the use of BTA in the acute setting to minimize scarring and reduce contracture formation.

**Methods:**

This is a case study on a 16-year-old male with a 48% TBSA full thickness flame burn from an electrical arc, most significantly to his torso and bilateral upper extremities. The patient underwent excisional debridement and allografting within the first 72 hours after injury. Seven days later, at the time of autografting, BTA was injected under ultrasound guidance into the bilateral sternocleidomastoid (20 units each), bilateral pectoralis major (20 units each), and platysma muscles (20 units) for a total of 100 units. He required two operations to complete his autografting. He resumed aggressive therapy with range of motion exercises after 5 days of immobilization following each grafting surgery.

**Results:**

The patient did not have any complications from BTA injections. He was able to actively participate in burn therapy over the entirety of his hospital course. Table 1 shows the bilateral shoulders’ active range of motion measurements after BTA injection. The photos in Figure 1 are representative of those measurements.

**Conclusions:**

A paucity of literature investigating the use of BTA in chronic burn scar contractures exists. To our knowledge, this is the first reported use of BTA in the management of acute burns to reduce or prevent contractures. This case study demonstrates safe administration of BTA. Drawing further conclusions of efficacy is challenging given the lack of standardized data collection or benchmarks for range of motion after burn injury. Prospective data will be beneficial in showing this efficacy and then subsequently identifying ideal patients and optimizing timing of administration.

**Applicability of Research to Practice:**

This report of the use of BTA injections in acute burns should increase the interest in research regarding its use in the treatment algorithm for initial grafting post-injury. This case report highlights the need for range of motion benchmarks for burn patients and survivors.